# Response of Microbial Communities to Changing Climate Conditions During Summer Cyanobacterial Blooms in the Baltic Sea

**DOI:** 10.3389/fmicb.2018.01562

**Published:** 2018-07-25

**Authors:** Christoffer Berner, Mireia Bertos-Fortis, Jarone Pinhassi, Catherine Legrand

**Affiliations:** Department of Biology and Environmental Science, Centre for Ecology and Evolution in Microbial Model Systems (EEMiS), Linnaeus University, Kalmar, Sweden

**Keywords:** microscopy, 16S rRNA, cyanobacteria, heterotrophic bacteria, biomass, summer bloom, Baltic Sea, climate change

## Abstract

Frequencies and biomass of Baltic Sea cyanobacterial blooms are expected to be higher in future climate conditions, but also of longer duration as a result of increased sea surface temperature. Concurrently, climate predictions indicate a reduced salinity in the Baltic Sea. These climate-driven changes are expected to alter not solely the phytoplankton community but also the role of microbial communities for nutrient remineralization. Here, we present the response of summer plankton communities (filamentous cyanobacteria, picocyanobacteria, and heterotrophic bacteria) to the interplay of increasing temperature (from 16 to 18°C and 20°C) and reduced salinity (from salinity 6.9 to 5.9) in the Baltic Proper (NW Gotland Sea) using a microcosm approach. Warmer temperatures led to an earlier peak of cyanobacterial biomass, while yields were reduced. These conditions caused a decrease of nitrogen-fixers (*Dolichospermum* sp.) biomass, while non nitrogen-fixers (*Pseudanabaena* sp.) increased. Salinity reduction did not affect cyanobacterial growth nor community composition. Among heterotrophic bacteria, *Actinobacteria* showed preference for high temperature, while *Gammaproteobacteria* thrived at *in situ* temperature. Heterotrophic bacteria community changed drastically at lower salinity and resembled communities at high temperature. Picocyanobacteria and heterotrophic bacterial biomass had a pronounced increase associated with the decay of filamentous cyanobacteria. This suggests that shifts in community composition of heterotrophic bacteria are influenced both directly by abiotic factors (temperature and salinity) and potentially indirectly by cyanobacteria. Our findings suggest that at warmer temperature, lower yield of photosynthetic cyanobacteria combined with lower proportion of nitrogen-fixers in the community could result in lower carbon export to the marine food web with consequences for the decomposer community of heterotrophic bacteria.

## Introduction

Interactions between photosynthetic phytoplankton and heterotrophic bacterioplankton (decomposers) largely affect the fate of primary production in most aquatic systems (Azam and Malfatti, 2007). These planktonic microbial communities play an essential role in biogeochemical cycles in aquatic ecosystems. Phytoplankton is the base of the food web, fuelling the entire ecosystem with particulate and dissolved organic matter (Biddanda and Benner, 1997). This organic matter is partly transferred to higher trophic levels through grazing, while 50% is decomposed and remineralized by heterotrophic bacteria (Cole, 1982). Additionally, some phytoplankton, such as diazotrophic cyanobacteria, are able to fix nitrogen fertilizing the waters when nitrogen is limited. Nitrogen fixation is an advantageous trait to outcompete other phytoplankton (Niemi, 1979). In the Baltic Sea, for example, filamentous cyanobacteria can form extensive blooms during summer when nitrogen (N) to phosphorus (P) ratio is low above thermocline (N:P <10; Niemi, 1979). These blooms are visible to the naked eye upon a threshold of 50–100 mg cm^-3^ cyanobacterial biomass in coastal and offshore waters (Kononen, 1992; Legrand et al., 2015). The Baltic Sea is one of the largest brackish water bodies in the world with a strong surface salinity gradient from north to south (Matthäus, 2006). Many organisms are unable to cope with these salinity conditions making the Baltic Sea a low-biodiversity ecosystem, exceptionally vulnerable to changes (Philippart et al., 2011). Among the most important threats, the constant increase of human activities in open and coastal areas, especially industrialized agriculture, has enhanced eutrophication in the Baltic Sea (Wulff et al., 2007) and led to the expansion of hypoxic zones in both deep and coastal areas (Zillén and Conley, 2010) with occasional release of phosphate to the pelagic zone (Vahtera et al., 2007). In these nutrient conditions, blooms of cyanobacteria are also possible due to the presence of nitrogen-fixing taxa such as the filamentous *Aphanizomenon* sp., *Dolichospermum* sp. (formerly *Anabaena*), and *Nodularia spumigena*. Among cyanobacterial blooming taxa in the Baltic Sea, *Dolichospermum* sp. and *Nodularia spumigena* produce toxins, microcystins, and nodularins respectively, that can accumulate in higher trophic levels and pose a health risk to domestic animals and humans (Mazur-Marzec et al., 2013). Single-celled cyanobacteria (picocyanobacteria) in the Baltic Sea waters are mainly represented by the genus *Synechococcus*, not nitrogen-fixing nor toxic, that occurs all year round and blooms upon the decline of filamentous cyanobacterial blooms in late summer (Andersson et al., 2010; Lindh et al., 2015b; Bertos-Fortis et al., 2016; Celepli et al., 2017). During summer in the Baltic Sea, filamentous and single-celled cyanobacteria represent the major contributors to primary and export production in the upper layer, above thermocline.

Until now, heterotrophic bacterial taxa associated with cyanobacterial blooms have been mainly described in freshwater systems, and the communities are commonly represented by *Proteobacteria*, *Bacteroidetes*, *Actinobacteria*, and *Verrucomicrobia* (e.g., Eiler and Bertilsson, 2004; Kolmonen et al., 2004; Louati et al., 2015). The dynamics of these associations differ depending on environmental conditions, geographic location (Eiler and Bertilsson, 2004), genus/species composition, and growth phase of the cyanobacterial bloom (Louati et al., 2015).

Microbial communities are highly dynamic and fluctuate over the course of different timespans (hours, days, months, and years; Bunse and Pinhassi, 2017). These patterns are predicted to face strong alterations as a consequence of climate-related changes. The Baltic Sea seems to be warming up faster than other oceans, with predictions pointing to increasing precipitation and river run-off, and as consequence a reduction in salinity is expected (Meier et al., 2014). The average sea surface temperatures will potentially increase from 2 to 5°C (HELCOM, 2013), and salinity will be reduced 2–2.5 units by the end of the century (Neumann et al., 2012). Rising sea surface temperature is likely to catalyze the expansion of cyanobacterial blooms worldwide (Paerl and Huisman, 2008) and trigger earlier and longer blooms at higher latitudes (Neumann et al., 2012; Paerl and Paul, 2012). In the Baltic Sea, significant temporal changes in seasonality of phytoplankton blooms have been detected associated with climate change, revealing that cyanobacterial blooms have a longer productive season and form surface accumulations 20 days earlier than 35 years ago (Kahru and Elmgren, 2014; Kahru et al., 2016). In addition, a recent study has shown that a reduction of 0.6 units in salinity might trigger the occurrence of toxic cyanobacteria during summer (Bertos-Fortis et al., 2016). Besides this, cyanobacteria in the Baltic Sea have shown to tolerate a wide range of salinities (Lehtimäki et al., 1997; Mazur-Marzec et al., 2005) and temperatures (Lehtimäki et al., 1997; Pliński and Jozwiak, 1999). Climate-driven changes are also thought to alter phytoplankton stoichiometry (higher carbon-to-nutrient ratio), providing poor-quality food for higher trophic levels (Van de Waal et al., 2009; De Senerpont Domis et al., 2014). The response of heterotrophic bacteria from the Baltic Sea has been investigated under environmental conditions related to climate change in experimental manipulations with temperature (Eiler et al., 2007; Sjöstedt et al., 2012a; Lindh et al., 2013; von Scheibner et al., 2014) and salinity (Langenheder et al., 2003; Kaartokallio et al., 2005; Sjöstedt et al., 2012b).

Overall, it is still uncertain how the synergy of temperature and salinity will affect cyanobacteria and their associated heterotrophic bacterial communities in the Baltic Proper, and to what extent these organisms will adapt. The aim of this study was to provide experimental evidence on the effect of increasing temperature (+2 and +4°C) and decreasing salinity (-1 salinity units) on: the onset and biomass development of cyanobacteria, and their stoichiometry; the community composition of both cyanobacteria and their associated heterotrophic bacteria. Microbial community composition was assessed using morphological/microscopical (filamentous cyanobacteria, picocyanobacteria, and heterotrophic bacteria) and molecular (all prokaryotes) determination.

## Materials and Methods

### Sample Collection and Microcosm Setup

Seawater used as inoculum for the experiment was collected on the 9th of July 2014 using a Ruttner sampler at the Linnaeus Microbial Observatory (LMO) situated 10 km off the east coast of Öland (Sweden) in the Baltic Sea Proper. Water was collected at 5 depths (2, 4, 6, 8, and 10 m) and pooled as a collective sample representative of the water column above the thermocline (10.1 m). Physical, chemical, and oceanographic profile data were measured for *in situ* conditions of the inoculum (LMO), including phytoplankton and bacterial community composition (Supplementary Figure 1 and Supplementary Table 1). In the upper mixed layer, *in situ* mean temperature and salinity were 16.5°C and 6.9, respectively. Dissolved inorganic nitrogen (DIN = NO_3_^-^+NH_4_^+^) and phosphate (PO_4_^-3^) concentrations were low as expected during summer, and N:P ratio was higher than Redfield ratio (>18; Supplementary Table 1). The natural phytoplankton community in the LMO station was dominated by cyanobacteria (>70% of the total phytoplankton biomass).

Unfiltered seawater was transported to the laboratory in acid washed, Milli-Q rinsed, polycarbonate bottles within 1 h. This seawater was kept 2 h in the dark at 16°C prior to be prepared for inoculum in experimental manipulations. The inoculum was prepared by filtering the seawater through a 200-μm mesh to remove large-sized predators and distributed equally (ratio of the inoculum 1:64, final concentration of chlorophyll *a* 0.67 ± 0.03 μg L^-1^) in 16 microcosms (25L) filled with sterile filtered (0.2 μm) seawater from the Kalmar Sound at 16, 18, and 20°C (acclimated overnight in constant temperature rooms) and manually mixed with a plastic disk. An additional treatment at 18°C was set reducing the initial salinity by one unit (from salinity 6.9 to 5.9) adding MQ-water [named 18°C(-S)]. Four replicates were set for each treatment, in total 16 microcosms. Kalmar Sound coastal water was selected as growth medium primarily because it had a low N:P ratio (∼5) and high DIN and PO_4_^-3^ concentrations, 2.54 and 0.47 μm, respectively, that would allow cyanobacterial proliferation (Supplementary Table 2). The microcosms were exposed to a 16:8 light/dark cycle at a light intensity of 120–130 μmol m^-2^ s^-1^ to mimic *in situ* irradiance levels in the upper mixed layer during summer in the Baltic Proper (measured on sampling day, Supplementary Figure 1). Microcosms were incubated for 23 days and were gently mixed manually every second or third day prior to sampling. The experiment was stopped when chlorophyll *a* levels declined. Phosphate (PO_4_^-3^), 0.1 μm, was added to treatments 18, 18(-S), and 20°C on day 17 to avoid limitation, as levels were below 0.2 μm. Samples for community composition (abundance/biomass/DNA) and other samples (inorganic nutrients/stoichiometry) were monitored at different intervals during the experiment, see details below.

### Dissolved Inorganic Nutrients

Samples for nitrate (NO_3_^-^), ammonia (NH_4_^+^), phosphate (PO_4_^-3^), and silica (Si) were measured on days 1 (initial), 9, 16, and 23. Samples were analyzed using colorimetric methods according to Valderrama (1995).

### Chlorophyll *a*, Cyanobacterial and Heterotrophic Bacterial Biomass

Biomass and abundance (Chl *a*, filamentous cyanobacteria, picocyanobacteria, and heterotrophic bacteria) were determined every second or third day. Chlorophyll *a* was measured fluorometrically on ethanol extracts (Jespersen and Christoffersen, 1987). Samples for filamentous cyanobacterial abundance and identification were preserved with 2% Lugol’s solution and kept in the dark until analysis. Subsamples were transferred into sedimentation chambers and left overnight before counting with an Olympus CKX 41 inverted light microscope. In each sample, at least 350 filaments were counted. Filaments were identified to the genus or species level when possible. Biovolumes (Olenina et al., 2006) were transformed to carbon content (Edler, 1979) to obtain cyanobacterial biomass. Samples for enumeration of picocyanobacteria and heterotrophic bacteria were preserved in 2% formaldehyde and kept at -80°C until further analysis. Samples were stained with SYBR gold and filtered through a 0.2 μm pore sized black polycarbonate filter (Nuclepore). Heterotrophic bacteria were enumerated under blue light excitation, and picocyanobacteria were detected by their red autofluorescence. Epifluorescence microscope (Olympus BX50) was used to count at least 300 cells for each sample. Picocyanobacterial counts showed that rod-shaped cells dominated the community regardless of treatments. Picocyanobacterial counts were converted to biomass using the conversion factor 0.8 pg C cell^-1^ (Olenina et al., 2006), and bacterial abundance was converted to biomass using a factor of 20 fg C cell^-1^ (Lee and Fuhrman, 1987). We consider these conversion factors as proxy to estimate averaged biomass obtained in the different treatments of the experiment. However, cell size variability among similar morphotypes can be significant in bacteria from both groups in the Baltic Sea (Albertano et al., 1997; Blackburn et al., 1998; Haverkamp et al., 2009), and we are aware of the limitations of our biomass estimations.

### Stoichiometry of the Community

Samples for particulate organic carbon (POC), nitrogen (PON), and phosphorus (POP) were taken for elemental composition determination on days 1, 9, 16, and 23. Aliquots of 700 mL (POC and PON) and 200 mL (POP) were filtered onto precombusted (450°C, 2 h) Whatman glass fiber carbon (GF/C) filters, dried at 60°C for 8 h and stored in a desiccator until further analysis. POC and PON filters were analyzed with a CHNS/O Analyzer (2400 Series II, PerkinElmer). POP filters were processed and measured according to the method of Salórzano and Sharp (1980). C:P, N:P, and C:N ratios were calculated on a molar basis.

### Collection, Extraction, and Sequence Analysis of DNA

Biomass for DNA extraction was collected on days 1, 9, 16, and 23. Water samples (1 L) from each replicate were filtered onto 0.2 μm 47 mm Supor filters (PALL Life Sciences). Filters were amended with TE buffer and immediately stored in -80°C until further processing. DNA was extracted using a phenol-chloroform protocol described by Boström et al. (2004). Bacterial 16S rRNA genes were amplified with the primers 341F and 805R containing adaptor and barcode (Herlemann et al., 2011) following Hugerth et al. (2014) PCR protocol with some modifications. Briefly, the first PCR was performed in duplicates for each biological replicate and we used an annealing temperature of 58°C. In the second PCR, 12 cycles were performed. The purified PCR amplicons were sequenced on the Illumina Miseq platform, at the Science for Life Laboratory, Stockholm, Sweden.

Raw sequence data generated from Illumina Miseq were analyzed using the UPARSE pipeline (Edgar, 2013). Sequences were stripped, merged, and quality-controlled according to default settings (Edgar, 2013) and clustered into operational taxonomic units (OTUs) at 97% identity, and singletons were removed. Taxonomy was determined using BLAST against the SILVA database, using the SINA aligner (Pruesse et al., 2012). After quality control, our data consisted of a total of 3 million reads, 656,133 raw reads of cyanobacteria, and 2,344,343 raw reads of heterotrophic bacteria. Samples with fewer than 1,800 reads were excluded from further analysis (i.e., day 1 replicate 2 and day 23 treatment 18°C(-S) replicates 1, 3, and 4). In total, 90 cyanobacterial OTUs and 1146 heterotrophic bacterial OTUs were identified. For the OUT-based analyses, chloroplast sequences were excluded. Normalization was carried out using the total-sum of reads per sample. The 16S rRNA gene sequences were deposited in the National Center for Biotechnology Information Sequence Read Archive under accession number SRP120581.

### Statistical Analysis

All statistical analyses were performed using RStudio Version 0.98.945. The relationship between Chl *a* and cyanobacterial biomass (filamentous cyanobacteria and picocyanobacteria) was studied using linear regression. Data were log-, square root, or Box-Cox transformed to meet the assumption of normality. To determine the effects of temperature and salinity on biomass (Chl *a*, filamentous cyanobacteria, picocyanobacteria, and heterotrophic bacteria) and stoichiometry of the community during the experiment, linear mixed models were performed. Time and temperature or time and salinity were set as fixed effects, while replicates within treatments were set as random effect. As on many occasions relationships with time were quadratic, 2nd degree polynomial functions were included in the models. The process of model selection was top-down and the package *nlme* was used (Pinheiro et al., 2017). Cumulative biomass was the sum of the different groups/genus biomass over the duration of the experiment. Differences between treatments of respective cumulative biomass of *Dolichospermum*, *Pseudanabaena*, and picocyanobacteria were tested with one-way ANOVA and *post hoc* Tukey test. Community composition of cyanobacteria (subset of cyanobacterial reads) and heterotrophic bacteria (subset of heterotrophic bacterial reads) were analyzed using Non-metric Dimensional Scaling (NMDS) and hierarchical cluster analysis (for heterotrophic bacteria) with a Bray-Curtis distance matrix calculated from relative abundance of OTUs (normalized reads). To test if there was any effect of increased temperature and reduced salinity on community composition, we performed PERMANOVA followed by pairwise comparison with Bonferroni’s adjustment. Richness (Chao 1 index) and diversity (Shannon index) of heterotrophic bacteria were calculated, after rarefying to 1,800 reads per sample, with *Vegan* package (Oksanen et al., 2013). Plots were created using *ggplot2* (Wickham, 2016).

## Results

### Microbial Community Biomass

**FIGURE 1:**
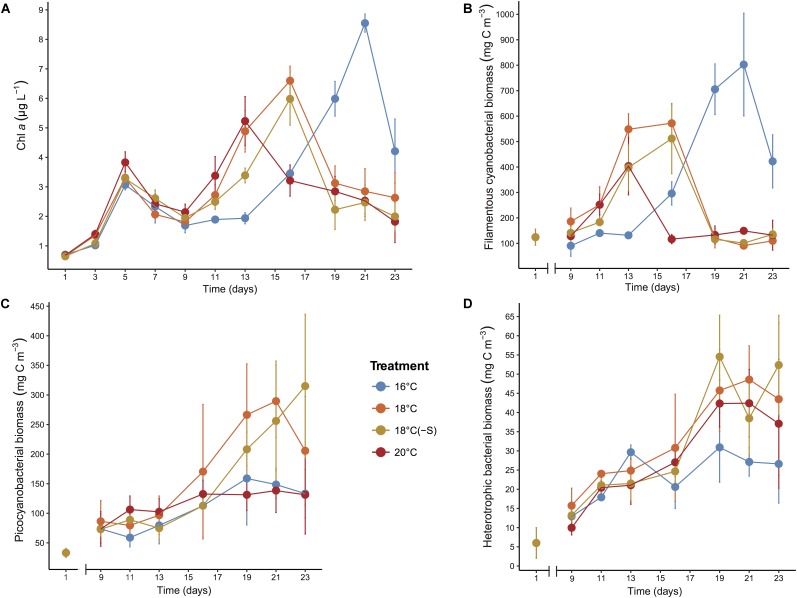
Development of **(A)** Chl *a*, **(B)** filamentous cyanobacterial biomass, **(C)** picocyanobacterial biomass, and **(D)** heterotrophic bacterial biomass during the microcosm experiments. Error bars denote SDs for replicate microcosms (*n* = 4 for Chl *a* concentration, *n* = 3 for biomass).

Chlorophyll *a* was a good proxy for cyanobacterial biomass during the experiment as they were highly correlated (*R*∼0.9; *p* < 0.001), therefore we can ensure that phytoplankton community was mainly composed of cyanobacteria. Phototrophic growth was similar from day 1 to 5 in all treatments, time of adaptation before passing the threshold of the bottle-effect of the incubation (**Figure 1A**). After that, maximum phytoplankton biomass was reached at different times for different treatments. Temporal evolution showed that filamentous cyanobacteria peaked earlier at 20°C (day 13) but with significantly lower biomass (548 ± 61 mg cm^-3^) compared to 18°C (day 16) and *in situ* temperature (16°C, day 21) (*F*_2,56_ = 5.96, *p* = 0.005; **Figure 1B**). Filamentous cyanobacterial biomass was highest at 16°C reaching 802 ± 201 mg cm^-3^ (*p* < 0.001). Picocyanobacterial biomass was highest in the 18°C treatments, reaching levels of ∼250 mg cm^-3^ on day 21 (**Figure 1C**). Cyanobacterial growth led to a reduction of DIN and PO_4_^-3^ concentrations in the microcosms during the experiment, maintaining nitrogen-limited conditions (N:P = 5–10; Supplementary Table 2). Heterotrophic bacterial biomass steadily increased from day 9 until day 19 and remained stable until the end of the experiment. A less pronounced development of heterotrophic bacteria biomass was detected at 16°C (**Figure 1D**), but no significant differences were detected between the treatments. Overall, lower salinity did not have any significant effect on bloom development, Chl *a* maximum, cyanobacterial biomass (filamentous and pico-cyanobacteria), nor heterotrophic bacterial biomass (*p >* 0.05, **Figure 1**).

### Community Stoichiometry

POC and PON concentrations followed the same pattern during the experiment (**Table 1**). While treatments at 18 and 20°C showed a higher peak on day 16, colder temperature treatments showed a maximum POC and PON concentration on day 23. POC and PON concentrations were significantly different between 16°C and warmer temperatures (POC: *F*_2,9_ = 6.87, *p* = 0.015; PON: *F*_2,9_ = 5.32, *p* = 0.029). POP concentrations increased until day 16 and then remained stable, and no significant difference was detected among treatments. The molar ratio of POC:PON decreased during the experiment in all treatments and was significantly lower in the 20°C microcosms throughout the experiment (*F*_2,9_ = 21.24, *p* < 0.01), but was not affected by salinity (**Table 1**). POC:POP, PON:POP, and POC:Chl *a* ratios were stable during the experiment, and no significant difference was detected between treatments of different temperatures nor salinity (*p* > 0.05).

**Table 1:** Stoichiometry of the community.

		16°C	18°C	18°C(-S)	20°C
POC (μM)	Day 1	12.0 ± 0.8	12.0 ± 0.8	12.0 ± 0.8	12.0 ± 0.8
	Day 9	21.4 ± 1.0	25.2 ± 3.7	26.6 ± 2.6	24.2 ± 2.2
	Day 16	33.6 ± 3.7	61.3 ± 3.1	49.2 ± 4.8	34.3 ± 3.7
	Day 23	57.1 ± 5.9	46.7 ± 7.3	44.9 ± 7.6	38.0 ± 7.6
PON (μM)	Day 1	1.37 ± 0.1	1.37 ± 0.1	1.37 ± 0.1	1.37 ± 0.1
	Day 9	2.35 ± 0.0	2.97 ± 0.4	3.12 ± 0.3	3.05 ± 0.2
	Day 16	4.72 ± 0.5	8.85 ± 0.4	7.10 ± 0.7	5.40 ± 0.6
	Day 23	8.30 ± 0.9	6.82 ± 0.6	6.26 ± 0.9	5.70 ± 0.9
POP (μM)	Day 1	0.03 ± 0.0	0.03 ± 0.0	0.03 ± 0.0	0.03 ± 0.0
	Day 9	0.07 ± 0.0	0.05 ± 0.0	0.08 ± 0.0	0.08 ± 0.0
	Day 16	0.15 ± 0.0	0.22 ± 0.0	0.13 ± 0.0	0.13 ± 0.0
	Day 23	0.13 ± 0.0	0.19 ± 0.0	0.11 ± 0.0	0.14 ± 0.0
POC:PON	Day 1	8.75 ± 0.1	8.75 ± 0.1	8.75 ± 0.1	8.75 ± 0.1
	Day 9	9.13 ± 0.4	8.50 ± 0.3	8.57 ± 0.5	7.94 ± 0.2
	Day 16	7.12 ± 0.0	6.92 ± 0.0	6.93 ± 0.2	6.36 ± 0.1
	Day 23	6.89 ± 0.1	6.82 ± 0.4	7.17 ± 0.4	6.64 ± 0.2
POC:POP	Day 1	384 ± 60	384 ± 60	384 ± 60	384 ± 60
	Day 9	351 ± 23	489 ± 17	348 ± 16	264 ± 22
	Day 16	252 ± 13	316 ± 17	391 ± 95	262 ± 76
	Day 23	492 ± 28	280 ± 13	408 ± 14	269 ± 98
PON:POP	Day 1	43 ± 6	43 ± 6	43 ± 6	43 ± 6
	Day 9	37 ± 23	58 ± 23	40 ± 17	33 ± 3
	Day 16	35 ± 18	45 ± 24	56 ± 16	41 ± 12
	Day 23	72 ± 44	40 ± 17	56 ± 17	40 ± 13
POC:Chl *a*	Day 1	17 ± 1.4	18 ± 1.8	18 ± 1.6	17 ± 1.4
	Day 9	12 ± 1.6	13 ± 1.8	13 ± 1.5	11 ± 1.8
	Day 16	9 ± 0.8	9 ± 0.4	8 ± 0.6	10 ± 0.9
	Day 23	14 ± 4.6	18 ± 4.5	22 ± 2.6	23 ± 9.1


*Concentrations of particulate organic C, N, P and POC:PON, POC:POP, PON:POP, POC:Chl *a* ratios in microcosms at 16, 18, 18(-S), and 20°C treatment (mean ± SD, *n* = 4).*

### Cyanobacterial Community Composition

**FIGURE 2:**
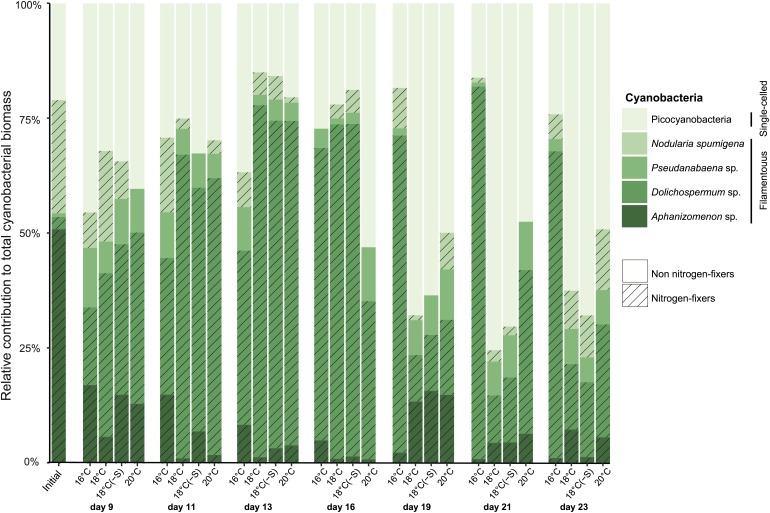
Averaged relative contribution of cyanobacterial taxa to the total cyanobacterial biomass over the microcosm experiments. Each bar represents a sampling date and treatment (*n* = 3).

**FIGURE 3:**
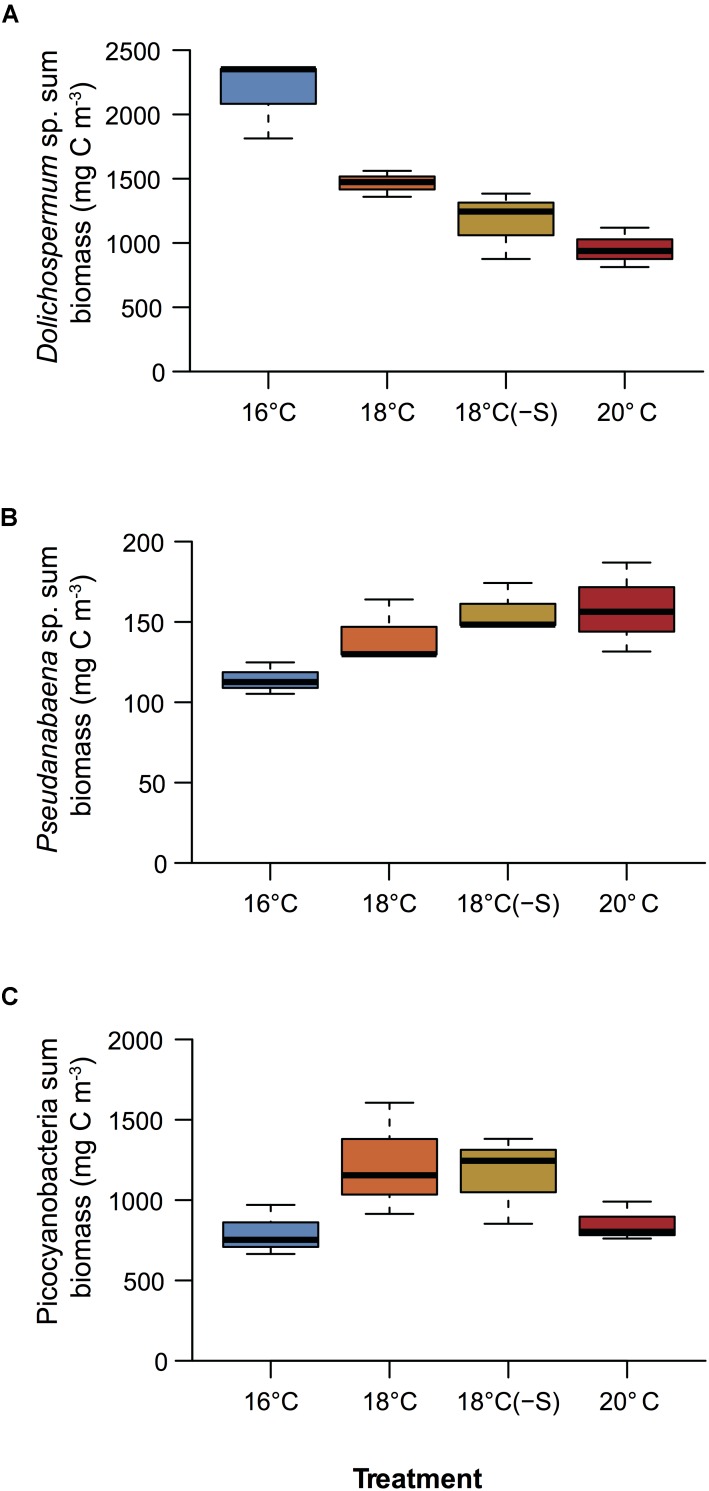
Cumulative biomass over the experimental period of **(A)** *Dolichospermum* sp., **(B)** *Pseudanabaena* sp., **(C)** and picocyanobacteria (*n* = 3). Colors correspond to treatments, see legend in **Figure 1**.

A total of 5 cyanobacterial taxa were identified by microscopy. Initially in the microcosms, the filamentous nitrogen-fixing *Aphanizomenon* sp. and *Nodularia spumigena* were the most abundant, reaching 51 and 25% of the total cyanobacteria biomass, respectively (**Figure 2**). After day 9, a third filamentous cyanobacterium, *Dolichospermum* sp. (previously classified as *Anabaena*), became the main contributor (>50% of total biomass) to the community in all treatments. The other dominant group of cyanobacteria was a mixture of the small and non nitrogen-fixing *Pseudanabaena* filaments and unicellular picocyanobacteria. Upon the decline of filamentous cyanobacterial biomass (end of the experiment), unicellular picocyanobacteria contributed the most to total cyanobacterial biomass in all treatments, specially at 18°C. Cumulative biomass of the most abundant filamentous nitrogen-fixing cyanobacteria, *Dolichospermum* and the non nitrogen-fixing *Pseudanabaena*, changed inversely with increasing temperature (**Figures 3A,B**). *Dolichospermum* biomass decreased (*F*_2,6_ = 28.6; *p* < 0.05), while *Pseudanabaena* biomass increased (*F*_2,6_ = 4.16; *p* = 0.07) in response to increasing temperature (**Figures 3A,B**). Salinity reduction did not have a significant effect on the cumulative biomass of the most abundant cyanobacteria (*p* > 0.05, **Figure 3**).

**FIGURE 4:**
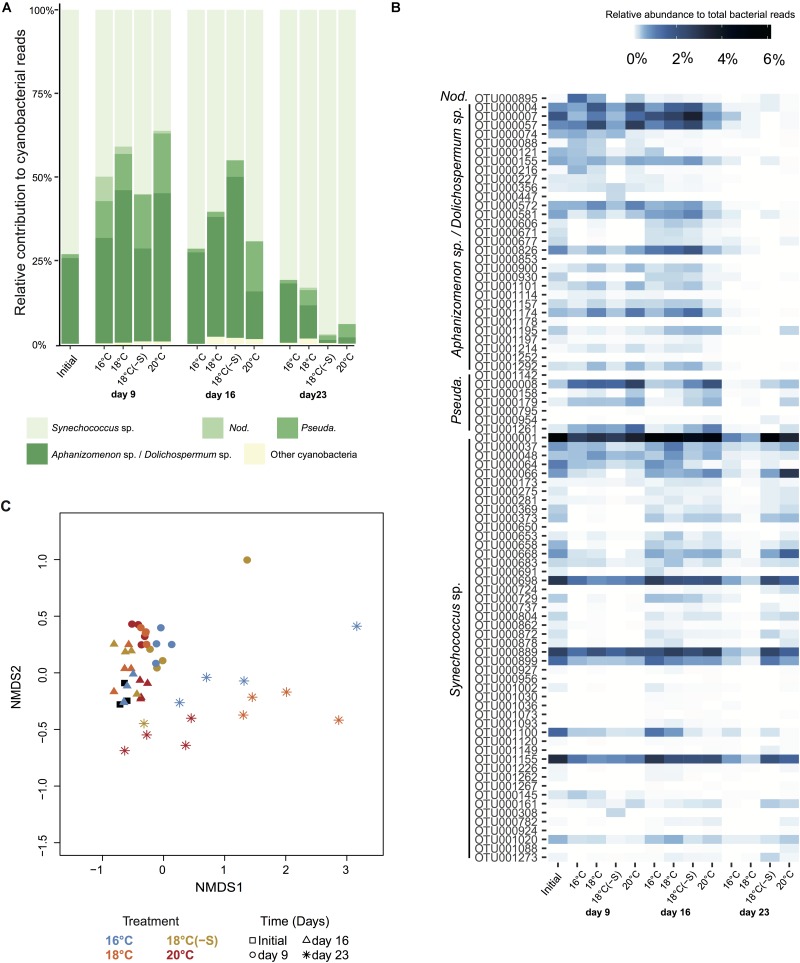
Cyanobacterial community composition at phylogenetic level. **(A)** Averaged relative contribution to cyanobacterial reads at 97% 16S rRNA gene identity. **(B)** Heatmap representing the averaged relative abundance to total bacterial reads of cyanobacterial OTUs. Abbreviations correspond to *Nodularia spumigena* (*Nod.*), and *Pseudanabaena* sp. (*Pseuda.*). **(C)** NMDS of bray Curtis dissimilarity matrix for cyanobacteria, stress = 0.06 (Initial *n* = 3; day 23 treatment 18°C(-S) *n* = 1; rest of the treatments and days *n* = 4). Colors indicate treatment and symbols indicate the day of the experiment.

Complementary to microscopy, the genetic composition of cyanobacteria can be assessed by analyzing the cyanobacterial sequences in the 16S rRNA gene libraries. Relative abundances of filamentous cyanobacterial sequences were a factor of 2–3 lower/higher compared to biomass proportions, and failed to unravel treatment responses such as high filamentous cyanobacterial abundance on day 23 at 16°C (**Figures 2**, **4A**). We identified 4 major distinct cyanobacterial genus/species with gene sequencing (**Figure 4A**), as *Aphanizomenon* and *Dolichospermum* cannot be differentiated due to their high similarity in the 16S rRNA gene. *Aphanizomenon/Dolichospermum* were abundant until day 16, reaching up to 50% of the total cyanobacterial reads (**Figure 4A**) and had the highest OTU number (30 OTUs) among filamentous cyanobacteria (**Figure 4B**). The most frequent *Aphanizomenon/Dolichospermum* OTUs (OTU000004, OTU000007, and OTU000057) were closely related to *Aphanizomenon/Dolichospermum* OTUs found in the southern Western Gotland Sea (99–100% similarity; OTU016419 and OTU000339; Bertos-Fortis et al., 2016; **Figure 4B**). *Nodularia spumigena* occurred mainly on day 9 and solely one OTU (OTU000895) was found in the experiment, being 99% similar to an isolate from the Baltic Sea (KF360088, Fewer et al., 2013) and also to an OTU found during summer in the southern Western Gotland Sea (OTU000113; Bertos-Fortis et al., 2016). Relative abundance of OTUs related to filamentous non nitrogen-fixing *Pseudanabaena* (7 OTUs) showed highest relative abundance at 20°C on days 9 and 16 (up to 17% of cyanobacterial sequences). Approximately, half of the cyanobacterial OTUs were identified as the picocyanobacterium *Synechococcus* sp (48 OTUs) (**Figure 4B**). *Synechococcus* OTUs were abundant throughout the experiment and in all treatments (up to 20% of total bacterial sequences and 95% of cyanobacterial reads). The dominant OTU (OTU000001) was 100% similar to a *Synechococcus* phylotype occurring all year round in the Central Baltic Proper (OTU000001; Bertos-Fortis et al., 2016; **Figure 4B**). The cyanobacterial community at OTU level clustered according to time of the experiment regardless of treatment (**Figure 4C**). Considering all days of the experiment, warming had an effect on the cyanobacterial community composition, while no effect was associated with lower salinity. As high dispersion was found among temperature treatment replicates, results should be interpreted with caution (PERMANOVA, Supplementary Table 3).

### Heterotrophic Bacterial Community Composition

**FIGURE 5:**
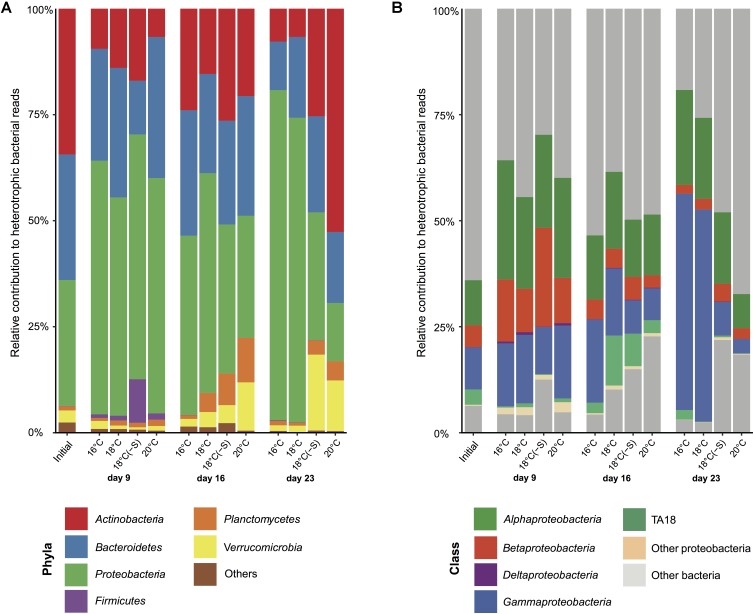
Heterotrophic bacterial community composition. Averaged heterotrophic bacterial reads at **(A)** phyla and **(B)** class level in microcosms at 16, 18, 18(-S), and 20°C treatments on day 1, 9, 16, and 23 (Initial *n* = 3; day 23 treatment 18°C(-S) *n* = 1; rest of the treatments and days *n* = 4).

For further information about the percentage of total bacterial sequences, including both cyanobacteria and heterotrophic bacteria, see Supplementary Figure S2. At the phyla level, initial bacterial community composition in the microcosms comprised 34% of *Actinobacteria*, 30% of *Bacteroidetes*, and 30% of *Proteobacteria* of heterotrophic bacterial reads (**Figure 5A**). Some changes in relative abundance of major phyla were detected by day 9, with a reduction of *Actinobacteria* (by 2–6 fold) and a doubling of *Proteobacteria*. On this day, heterotrophic communities at different temperatures were similar, but the community composition in the reduced salinity treatment had shifted somewhat and consisted of a higher relative abundance of *Firmicutes* at the expense of *Bacteroidetes* (**Figure 5A**). On day 16 onward, the relative abundance of *Actinobacteria* had increased compared to day 9. This increase continued at 20°C on day 23, while *Proteobacteria* increment was associated with 16 and 18°C. On day 23, the most abundant class of *Proteobacteria* at 16 and 18°C was *Gammaproteobacteria* (up to 51%; **Figure 5B**).

**FIGURE 6:**
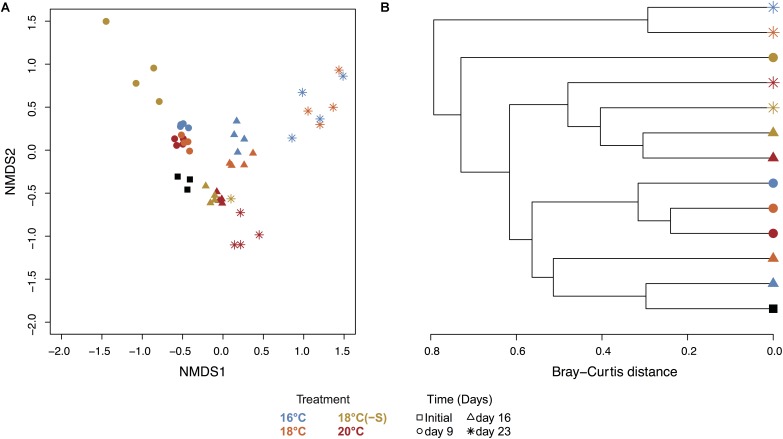
NMDS and cluster analysis of the heterotrophic bacterial community. **(A)** NMDS of heterotrophic bacterial community calculated from Bray-Curtis using 97% clustering, 16S rRNA (stress = 0.07, all replicates). **(B)** Hierarchical clustering based on Bray-Curtis distance between average of treatments. Colors indicate treatment and symbols indicate the day of the experiment (Initial *n* = 3; day 23 treatment 18°C(-S) *n* = 1; rest of the treatments and days *n* = 4).

Toward the end of the experiment (day 16 and day 23), heterotrophic communities clustered in two groups: (a) treatment 16 and 18°C and (b) treatment 18(-S) and 20°C (**Figure 6**). Significant differences in bacterial community composition could be observed between temperature and salinity treatments considering time (PERMANOVA, Supplementary Table 3). In all treatments, there were pronounced temporal changes in heterotrophic bacterial community composition, and up to 42% of the sums of squares in the linear mixed model could be explained by time. Moreover, analysis of the alpha diversity (Shannon index) revealed higher diversity at 18(-S) and 20°C (>4.4) compared to the other treatments (<3.5) on day 23 (**Table 2**). Richness (Chao1) levels were highest at 18(-S) and 20°C treatments on day 23 (**Table 2**).

**Table 2:** Richness and diversity of heterotrophic bacteria.

		16°C	18°C	18°C(-S)	20°C
Chao	Day 1	278 ± 10	278 ± 10	278 ± 10	278 ± 10
	Day 9	340 ± 26	353 ± 7	177 ± 43	364 ± 29
	Day 16	306 ± 37	356 ± 31	314 ± 23	340 ± 8
	Day 23	226 ± 48	219 ± 53	337 ± NA	359 ± 51
Shannon	Day 1	4.46 ± 0.29	4.46 ± 0.29	4.46 ± 0.29	4.46 ± 0.29
	Day 9	4.71 ± 0.07	4.88 ± 0.16	4.35 ± 0.27	4.79 ± 0.08
	Day 16	4.31 ± 0.31	4.61 ± 0.20	4.46 ± 0.08	4.74 ± 0.02
	Day 23	3.16 ± 0.52	3.01 ± 0.41	4.57 ± NA	4.45 ± 0.12


*Chao and Shannon index of heterotrophic bacteria during the experiment at 1,800 reads per sample (mean ± SD: Initial *n* = 3; day 23 treatment 18°C(-S) *n* = 1; rest of the treatments and days *n* = 4).*

**FIGURE 7:**
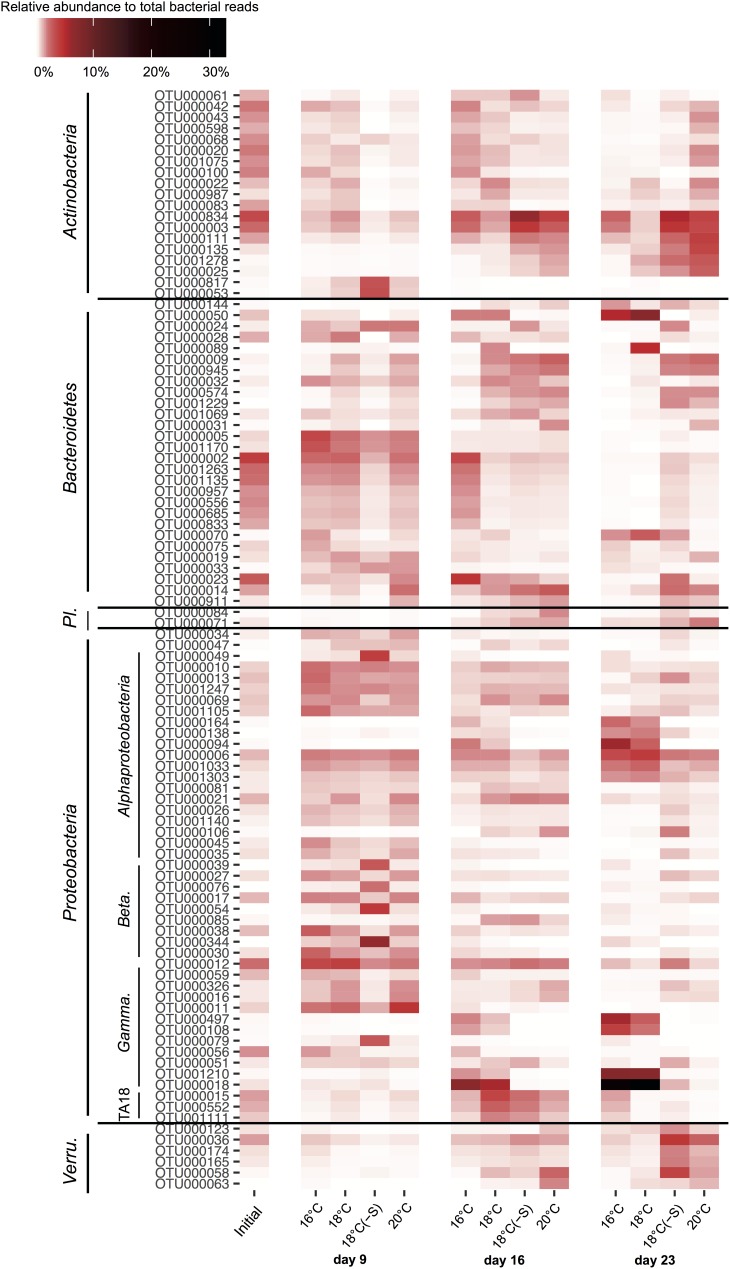
Dynamics of the 100 most abundant OTUs of heterotrophic bacteria during the experiment. The colors in the heatmap represent the averaged relative abundance of each OTU in a specific treatment and day (Initial *n* = 3; day 23 treatment 18°C(-S) *n* = 1; rest of the treatments and days *n* = 4). Abbreviations correspond to *Planctomycetes* (*Pl.*), *Betaproteobacteria* (*Beta.*), *Gammaproteobacteria* (*Gamma.*) and *Verrucomicrobia* (*Verru.*).

The most abundant actinobacterial OTUs at 18(-S) and 20°C (OTU000834, OTU000111, and OTU000003) were closely related to *Candidatus Aquiluna*, previously found in eutrophic ponds in tropical zones (97–98% identity; NR_125489.1; Hahn et al., 2004). Among bacteroidetes OTUs, different patterns of occurrence were detected. For example, OTU000050 occurred mostly at 16 and 18°C, while OTU000002 was present in all treatments throughout the experiment (**Figure 7**). They are identified as *Algoriphagus aquatilis* isolated from a freshwater lake (98%; Liu et al., 2009) and *Cellulophaga* from seawater (94%; Kahng et al., 2009), respectively. Importantly, a set of proteobacterial OTUs were essentially restricted to the 16 and 18°C treatments on days 16 and 23, where they reached high abundance. Their increase was especially pronounced on day 23; the alphaproteobacterial OTU000164, OTU000138, OTU000094, and the gammaproteobacterial OTU000497, OTU000108, OTU001210, OTU000018 (**Figure 7**). These alphaproteobaterial OTUs were 99% similar to *Albimonas donghaensis*, *Seohaeicola saemankumensis*, and *Citreicella marina*, respectively (NR_043685.1, Lim et al., 2008; NR_044437.1, Yoon et al., 2009; KC534395.1, Rajasabapathy et al., 2014). The first two gammaproteobacteria OTUs belonged to the order of *Alteromonadales* (*Idiomarina* sp.), and the others were identified as *Pseudomonadales* (99% identity *Pseudomonas stutzeri*; KU601282.1).

## Discussion

### The Effect of Temperature on Filamentous Cyanobacteria

The occurrence of diazotrophic cyanobacterial blooms is triggered by strong stratification in the water column, high nutrient availability (mainly P), and warmer temperatures (Niemi, 1979). In the Baltic Proper (Central Baltic Sea), cyanobacteria proliferate massively when sea surface temperature exceeds 18°C (Pliński and Jozwiak, 1999). There is a clear trend that average temperature will surpass this threshold during extended summer periods by 2,100 (Neumann et al., 2012). Multi-model simulations predict that by the end of this century, the first day of cyanobacterial occurrence in the Baltic Proper will be 20 days earlier compared to the current scenario (Neumann et al., 2012). In fact, short-term experiments (indoor mesocosms) show that already 1°C increase in water temperature resulted in a 1.5–2 days earlier development of spring bloom communities (Sommer et al., 2007; Hoppe et al., 2008; von Scheibner et al., 2014). Our findings indicate that similar trends can be detected in microcosms with summer communities dominated by bloom forming cyanobacteria from the Baltic Proper. Moreover, here we provide experimental evidence that such changes in temperature (from *in situ* to +4°C) may be critical for the onset of cyanobacterial blooms.

There is a broad consensus that global warming will intensify cyanobacterial blooms worldwide (Paerl and Huisman, 2008; O’Neil et al., 2012; Paerl and Paul, 2012; Hense et al., 2013). However, our study showed that cyanobacterial biomass rather is reduced by half at warmer temperatures compared to *in situ* temperatures. Autecological preferences of different species of cyanobacteria that co-occur in blooms are species-specific regarding temperature and irradiance (Eigemann et al., 2018), salinity (Lehtimäki et al., 1997; Mazur-Marzec et al., 2005), and nutrient acquisition (Olli et al., 2015). *Dolichospermum*, a large filament, for example thrives at lower temperature (16°C) compared to other species (Eigemann et al., 2018, this study). *Pseudanabaena* is abundant at warmer temperature but is a small form (cells are up to 30 times smaller than *Dolichospermum*), hence likely to contribute less to the total phytoplankton biomass. These findings suggest the importance of the initial microbial community composition and their species-specific niche differences to understand and model the community response to higher temperature in combination with other limiting resources.

At a global scale, changes in phytoplankton composition are seen in parallel with a decline of phytoplankton biomass in the oceans (IPCC, 2013). These trends have already been detected during the last century at global (Boyce et al., 2010) and local scale such as the North Atlantic Ocean (Morán et al., 2010) and the Baltic Sea (Müren et al., 2005; Sommer et al., 2007; Legrand et al., 2015). At warmer temperatures, the alteration of cyanobacterial biomass and composition could lead to a change of ecosystem services e.g., nitrogen supply in the Baltic Proper. Increasing temperature could lead the current community composition of nitrogen-fixing cyanobacteria to change toward non nitrogen-fixing genera, including *Pseudanabaena* among others (Klawonn et al., 2016; Paerl and Otten, 2016; this study). Considering that other primary producers, such as picocyanobacteria, and heterotrophic bacteria use on average 20–30% of the fixed nitrogen as ammonium (Ploug et al., 2011), a change to non nitrogen-fixers might imply a reduction of primary and secondary production. On the other hand, a reduction of *Dolichospermum* sp. might be beneficial for aquatic organisms, as members of this genus produce hepatotoxins (microcystin) and other biologically active compounds (Halinen et al., 2007; Mazur-Marzec et al., 2015) that potentially negatively affect a wide range of trophic levels (e.g., Karjalainen et al., 2007).

Temperature, together with nutrient supply ratios and plankton community structure, is the main factor influencing elemental stoichiometry (Martiny et al., 2013). Field studies have shown that cyanobacteria from the Baltic Sea have higher C:P (200–400) and N:P (25–60) ratios but a lower C:N (6–7) ratio compared to Redfield ratios (Walve and Larsson, 2010). In our experiment, the planktonic microbial community was mainly composed of cyanobacteria (∼90% of the total phytoplankton biomass), and similar ratios were obtained except for C:N ratios (**Table 1**). High C:N ratios, in our study with a summer community, highlight that nitrogen was the most limiting nutrient toward the end of the experiment. In addition, results on community stoichiometry in different treatments revealed that C:N ratios were lowest at warmest temperature. These findings are rather different from previous studies showing that warmer temperatures have the potential to increase primary production and carbon export relative to the amount of growth-limiting nutrient (C:N ratio) (Finkel et al., 2010 and references therein). This suggests that the stoichiometry of cyanobacterial blooms from the brackish Baltic Sea might respond differently to climate-driven changes compared to cyanobacteria in other environments, and also other phytoplankton populations. Whether warmer temperatures can shift to a reduction of carbon export during summer blooms remains to be tested.

### Picocyanobacterial Occurrence

At the onset of the experiment, diazotrophs first occurred (*Aphanizomenon* sp., *Dolichospermum* sp., *Nodularia spumigena*) followed by an increase in biomass of non nitrogen-fixing picocyanobacteria (**Figure 1**). Analysis of nanoSIMS (high resolution imaging mass spectrometry) demonstrates that Baltic picocyanobacteria are unable to fix nitrogen (Karlson et al., 2015), suggesting that picocyanobacterial growth could be dependent mainly on the pulse of bioavailable nitrogen released by filamentous nitrogen-fixing cyanobacteria during summer (Ohlendieck et al., 2000; Ploug et al., 2011). These successional patterns of nitrogen-fixing cyanobacteria and picocyanobacteria have also been found in different parts of the Baltic Sea using morphological or molecular approach (Ohlendieck et al., 2000; Mazur-Marzec et al., 2013; Bertos-Fortis et al., 2016). Field studies have also shown that maximum picocyanobacterial biomass occurs at surface temperature of 17–18°C (Andersson et al., 1994; Mazur-Marzec et al., 2013). Taken together, these findings provide further evidence of the temporal associations between filamentous cyanobacteria and picocyanobacteria.

### Cyanobacteria at a Phylogenetic Level

Many cyanobacterial phylotypes reported to be abundant in the Baltic Proper (Western Gotland Sea) in field studies (Bertos-Fortis et al., 2016) were also abundant in our experiment. We found that the overall cyanobacterial community was slightly affected by temperature and salinity (**Figure 4C**). However, when considering individual populations (OTUs), a higher differentiation could be detected. Picocyanobacteria phylotypes showed two distinct occurrence patterns: some *Synechococcus* OTUs were present throughout the experiment at different temperatures and salinities (e.g., OTU000001), while others were the dominant forms at highest temperature at the end of the experiment (OTU000064). Filamentous cyanobacterial OTUs, on the other hand, had no clear preferences for temperature nor salinity treatments, although some *Pseudanabaena* OTUs (OTU000008 and OTU001261) were more pronounced at highest temperatures. These distinctive patterns among groups/phyla of both filamentous and picocyanobacteria were reported before in field studies (Andersson et al., 2010; Lindh et al., 2015b; Bertos-Fortis et al., 2016; Celepli et al., 2017).

Note that in our experiment, we found a mismatch between biomass proportion of cyanobacterial (microscopic counts) and relative abundance of sequences (16S rRNA) (**Figures 2**, **4A**). It is likely that the use of polyphasic approach is not commonly reported due to problems in interpreting dissimilar results. To our knowledge, only two studies have reported seemingly controversial issues, with cyanobacteria in the Western Gotland Sea (Baltic Proper) (Bertos-Fortis et al., 2016), and with other phytoplankton groups in multiple freshwater lakes (Eiler et al., 2013). Furthermore, Eiler et al. (2013) detected a phylogenetic group that could not be linked with any microscopic counts. In our study, *Aphanizomenon* sp. and *Dolichospermum* sp. were differentiated by microscopy due to their different morphology but their phylogeny is >99% similar for 16S rRNA (e.g., Gugger et al., 2002). Therefore, if we would have used solely one approach, relevant data would not have been unraveled. Conflicting results between microscopic and molecular analysis of *Synechococcus* were also observed utilizing DGGE profiles (Kolmonen et al., 2004). However, the comparison between DGGE profiles and 16S rRNA gene segments sequence analysis is rather problematic as the values of species richness are highly different between methods. In a single DGGE band, more than one species may be hidden resulting in underestimation of bacterial/cyanobacterial diversity. Ideally, number of cells should be comparable to gene copy numbers, assuming one 16S rRNA copy per cell. Recently, *Synechococcus* has been shown to have 1 or 2 copies of the 16S rRNA and this would explain why the two methodologies do not agree (Schirrmeister et al., 2012). Therefore, approaches targeting the 16S rRNA gene are not recommended as biomass indicator, but as a tool to describe the dynamics of cyanobacteria at a phylogenetic level. While it can be beneficial that molecular tools are not dependent of direct observations, it can be a major limitation as there can be multiple phylotypes for the same morphotype. Taken all together, although microscopy and molecular approaches have their shortcomings, they are complementary when used simultaneously. Furthermore, the polyphasic approach would be paramount for a better characterization of the diversity of cyanobacterial communities.

### Responses of Bacterial Biomass and Community Composition

Temperature is known to play an essential role in the regulation of microbial metabolism, i.e., influencing both growth rates and biomass production (Ducklow et al., 2010). In our study, the increase in heterotrophic bacterial bulk biomass was not significantly affected by temperature, and other microcosms and mesocosms studies have reported similar results both in relation to the spring bloom (Hoppe et al., 2008; Lindh et al., 2013; Bergen et al., 2016) and the summer bloom (Bergen et al., 2016). During our experiment, additionally, we could detect the largest increase of heterotrophic bacterial biomass when filamentous cyanobacteria decayed. A closer look to the field data presented by Bertos-Fortis et al. (2016) reveal the same pattern over different years and stations in the Western Gotland Sea. Our results indicate the potential of bacteria to maintain their biomass regardless of temperature, at least in the higher range. Moreover, the observed dynamics indicate that organic matter from filamentous cyanobacteria is an attractive food source for heterotrophic bacteria. This coupling between heterotrophic bacterial biomass and filamentous cyanobacteria is likely to have an important role on nutrient recycling for sustaining primary and secondary production in the Baltic Proper ecosystem.

The bacterial community composition in this study was dominated by phylogenetic groups commonly found in the Baltic Proper (Riemann et al., 2008; Andersson et al., 2010; Lindh et al., 2015b). Some OTUs described as *Bacteroidetes*, *Alphaproteobacteria*, and *Gammaproteobacteria* were stimulated by *in situ* temperature and +2°C. *Gammaproteobacteria*, showed similar trends in a microcosm experiment with Baltic Proper microbial communities during summer (Lindh et al., 2015a), indicating that they can grow at 16°C. The most abundant OTUs among *Alphaproteobacteria* in our experiment belonged to the *Rhodobacteraceae* family and have been found previously in Baltic Proper waters but were not present in the Bothnian Sea (Lindh et al., 2015a). *Verrucomicrobia* and *Actinobacteria* increased in abundance in response to +4°C. These phylogenetic groups are usually associated with freshwaters but are also found in brackish waters such as the Baltic Sea (Riemann et al., 2008; Andersson et al., 2010). *Actinobacteria* abundance has been associated with increasing temperatures in previous studies from the Baltic Sea (Holmfeldt et al., 2009), and this phylum is commonly found in late summer/autumn when temperature is elevated (Hugerth et al., 2015; Lindh et al., 2015b). In our study, the salinity reduction of one unit caused changes in heterotrophic bacterial community. Salinity plays an essential role in shaping community of heterotrophic bacteria in the Baltic Sea (Herlemann et al., 2011; Dupont et al., 2014), and experiments have confirmed these field observations (Langenheder et al., 2003; Kaartokallio et al., 2005; Sjöstedt et al., 2012b). However, in those studies, experimental evidence was obtained with unlikely high salinity changes (>5 salinity units) compared to current or future estimated salinity conditions, which limits extrapolations. In our study, even one-unit reduction of salinity caused pronounced changes for the heterotrophic community, thus indicating a likely change in the community function and composition under future climate conditions. Interestingly, OTUs frequently observed at lower salinity treatments showed also high relative abundance at +4°C. This indicated that both at community and OTU level, heterotrophic bacteria were adaptive to +2°C (18°C) but further temperature increase (20°C) and salinity decrease (1 unit) induced a substantial shift. Therefore, our study highlights that both temperature and salinity have major effects on summer heterotrophic bacterial community, with specific taxa being favored at certain conditions.

Bacterial community composition is not solely affected by abiotic factors such as temperature and salinity, but also by bioactive substances (e.g., allelochemicals and toxins) produced by the plankton community. Indirectly, substrates derived from phytoplankton might change due to experimental disturbances (e.g., temperature and salinity) and can provide distinct resource-based ecological niches benefiting specific bacterial taxa or populations (Pinhassi et al., 2004; Teeling et al., 2012). In our study, *Gammaproteobacteria* were the dominant forms (>50%) toward the end of the experiment, when the cyanobacterial bloom already had declined. *Gammaproteobacteria* are specialized in initial decomposition of algal-derived organic matter (Teeling et al., 2012), explaining their high occurrence in these conditions. In particular, the most abundant gammaproteobacterial OTUs were classified as *Pseudomonas stutzeri*. This bacterium has been found attached to cyanobacterial heterocysts, cells specialized in nitrogen fixation (Lupton and Marshall, 1981), and is a highly efficient denitrifier (Lalucat et al., 2006). Therefore, *Pseudomonas stutzeri* may play an important role in the nitrogen cycling at *in situ* temperature, fuelling the system with nitrogen both during and after the cyanobacterial bloom. On the other hand, in microcosms where low biomass (<100 mg C m^-3^) of filamentous cyanobacteria was maintained for several days, the relative abundance of *Actinobacteria* increased (**Figures 1B**, **5A**). A similar pattern has been observed in different locations in the Baltic Proper (Riemann et al., 2008; Hugerth et al., 2015; Lindh et al., 2015b). Recent studies have reported that *Actinobacteria* were associated with the utilization of labile carbon and nitrogen compounds (Lauro et al., 2011) and inversely related to cyanobacterial biomass in freshwater (Ghai et al., 2014). Particular genes have also been found encoding for degradation of both odd-carbon fatty acids and the storage compound cyanophycin (Ghai et al., 2014), further indicating a close connection between *Actinobacteria* and cyanobacterial blooms.

## Conclusion

Our study revealed that the increase in temperature predicted for future climate conditions (+2 and +4°C) may be critical for the timing of onset of cyanobacterial blooms. However, warmer temperature may not directly lead to higher primary production nor higher carbon, when cyanobacteria are the dominant photosynthetic microorganisms. This is the first experimental study describing the temporal succession of picocyanobacteria after a diazotrophic cyanobacterial bloom decay. Cyanobacterial phylotypes and the succession between nitrogen-fixers and non nitrogen-fixers observed in experimental conditions, were representative of those found in the Baltic Proper, indicating their potential to thrive under future climate-induced changes. At a genetic level, responses of cyanobacteria community to temperature and salinity treatments were more resilient than those of heterotrophic bacteria. Furthermore, this study emphasized the need to unravel both at chemical and molecular level the associations between photosynthetic cyanobacteria with specific groups of heterotrophic bacteria, as they play a major role in biogeochemical cycles. Highlighting the complex response of pelagic microbial communities to climate change, our results emphasize that cyanobacterial bloom onset and intensity, and environmental conditions will influence the taxa dominating the bacterial community composition, and probably their function.

## Author Contributions

CL and JP conceived the study and helped with data interpretation. CB, MB-F, and CL designed the research and wrote the manuscript. CB and MB-F performed the sampling. CB performed cyanobacterial counting and molecular work. MB-F and CB analyzed the data. All authors discussed the results and commented on the manuscript.

## Conflict of Interest Statement

The authors declare that the research was conducted in the absence of any commercial or financial relationships that could be construed as a potential conflict of interest.
