# Supplementary material for: Response of Microbial Communities to Changing Climate Conditions During Summer Cyanobacterial Blooms in the Baltic Sea
Source: Front Microbiol. 2018 Jul 25;9:1562. doi: 10.3389/fmicb.2018.01562 (PMC6068395; doi:10.3389/fmicb.2018.01562)
Supplement: Supplementary file 1 [file Data_Sheet_1.docx]

Supplementary Material

**Response of microbial communities to changing climate conditions during summer cyanobacterial blooms in the Baltic Sea**

**Christoffer Berner^1^, Mireia Bertos-Fortis^1^, Jarone Pinhassi^1^, Catherine Legrand^1*^**

^1^ Department of Biology and Environmental Science, Centre for Ecology and Evolution in Microbial Model Systems, Linnaeus University, Kalmar, Sweden.

*Correspondence: Catherine Legrand, catherine.legrand@lnu.se

## Supplementary Figures

**Supplementary Figure 1. Oceanographic profile collected on the 9^th^ July 2014 in Linnaeus Microbial Observatory (LMO) station.**

**Supplementary Figure 2. Overall bacterial community composition.** Averaged cyanobacterial and heterotrophic bacterial community composition in microcosms at 16°C, 18°C, 18°C(-S) and 20°C treatments on days 1, 9, 16, and 23 (Initial *n* = 3; day 23 treatment 18◦C(-S) *n* = 1; rest of the treatments and days *n* = 4).

1. **Supplementary Tables**

**Supplementary Table 1.** *In situ* data (A) seawater physical and chemical properties (B) phytoplankton community composition (biomass) and **(C)** prokaryotic community composition (16S rRNA) at Linnaeus Microbial Observatory (LMO) station at 2m depth on the 9^th^ July 2014.

| A |  |  |  | B |  |  |  | C |  |  |
| --- | --- | --- | --- | --- | --- | --- | --- | --- | --- | --- |
|  | **Temperature** | 17.8°C |  |  | **Chl *a*** | 2.95 µg L^-1^ |  |  | **Actinobacteria** | 2.0% |
|  | **Salinity** | 6.9 |  |  | **Diatoms** | 11.3 mg C m^-3^ |  |  | **Bacteroidetes** | 1.5% |
|  | **pH** | 8.42 |  |  | **Dinoflagellates** | 20.5 mg C m^-3^ |  |  | **Cyanobacteria** | 89.0% |
|  | **DIN** | 0.56 µM |  |  | **Cyanobacteria** | 244.1 mg C m^-3^ |  |  | **Planctomycetes** | 0.6% |
|  | **PO_4_^-3^** | 0.03 µM |  |  | **Golden algae** | 0 mg C m^-3^ |  |  | **Proteobacteria** | 6.0% |
|  | **N:P** | 18.7 |  |  | **Small flagellates** | 73.2 mg C m^-3^ |  |  | **Others** | 0.9% |
|  | **Silicate** | 11.80 µM |  |  |  |  |  |  |  |  |

**Supplementary Table 2. Inorganic nutrient development during the experiment.** Evolution of dissolved inorganic nutrients (μM) at 16°C, 18°C, 18°C (-S) and 20°C throughout the experiment (mean ± SD, *n*=4). Dissolved inorganic nitrogen (DIN) is the sum of nitrogen (NO_3_^-^) and ammonium (NH_4_^+^).

|  |  | **16°C** | **18°C** | **18°C (-S)** | **20°C** |
| --- | --- | --- | --- | --- | --- |
| **DIN** (μM) | Day 1 | 2.54±0.27 | 2.54±0.27 | 2.54±0.27 | 2.54±0.27 |
|  | Day 9 | 0.74±0.07 | 0.82±0.10 | 0.70±0.09 | 0.65±0.03 |
|  | Day 16 | 1.14±0.28 | 0.98±0.28 | 1.20±0.24 | 1.09±0.14 |
|  | Day 23 | 1.86±0.48 | 1.92±0.43 | 1.14±0.28 | 1.64±0.12 |
| **PO_4_^-3^** (μM) | Day 1 | 0.47±0.04 | 0.47±0.05 | 0.47±0.05 | 0.49±0.03 |
|  | Day 9 | 0.32±0.03 | 0.36±0.07 | 0.23±0.04 | 0.29±0.02 |
|  | Day 16 | 0.26±0.01 | 0.20±0.02 | 0.16±0.04 | 0.17±0.04 |
|  | Day 23 | 0.25±0.01 | 0.18±0.01 | 0.14±0.03 | 0.16±0.04 |
| **N:P** (μM) | Day 1 | 5.36±0.80 | 5.34±0.54 | 5.45±0.83 | 5.13±0.73 |
|  | Day 9 | 2.29±0.34 | 2.37±0.87 | 3.09±0.90 | 2.23±0.27 |
|  | Day 16 | 4.32±1.30 | 5.08±2.03 | 8.36±4.25 | 6.48±0.90 |
|  | Day 23 | 7.25±1.76 | 10.3±2.56 | 8.06±2.22 | 10.5±3.29 |
| **Si** (μM) | Day 1 | 13.23±1.08 | 13.23±1.08 | 13.23±1.08 | 13.23±1.08 |
|  | Day 9 | 8.06±0.86 | 8.13±1.50 | 9.45±1.97 | 11.9±1.90 |
|  | Day 16 | 8.81±1.25 | 8.98±0.97 | 8.05±0.31 | 9.03±0.28 |
|  | Day 23 | 6.70±0.32 | 3.67±1.07 | 4.52±1.10 | 1.64±0.51 |

**Supplementary Table 3. PERMANOVA results comparing the community composition of (A) cyanobacteria and (B) heterotrophic bacterial community at different temperature and salinity treatments.** Abbreviations correspond to temperature (Temp.) and salinity (Salinity).

| **A** Cyanobacterial community | | | | |  |  |  |  |  |  |  |  |  |  |
| --- | --- | --- | --- | --- | --- | --- | --- | --- | --- | --- | --- | --- | --- | --- |
|  |  |  |  |  |  |  |  |  |  |  |  |  |  |  |
|  |  | df | SS | *F* | R^2^ | *p* |  |  |  | df | SS | *F* | R^2^ | *p* |
|  | Time | 2 | 2.88 | 19.61 | 0.44 | **0.001** |  |  | Time | 2 | 1.38 | 6.56 | 0.35 | **0.001** |
|  | Temp. | 2 | 0.73 | 4.98 | 0.11 | **0.001** |  |  | Sal. | 1 | 0.16 | 1.54 | 0.04 | 0.16 |
|  | Time*Temp. | 4 | 0.95 | 3.25 | 0.15 | **0.018** |  |  | Time*Sal. | 2 | 0.83 | 3.93 | 0.21 | **0.007** |
|  | Residuals | 27 | 0.07 |  | 0.3 |  |  |  | Residuals | 15 | 1.58 |  | 0.4 |  |
|  |  |  |  |  |  |  |  |  |  |  |  |  |  |  |
| **B** Bacterial community | | | |  |  |  |  |  |  |  |  |  |  |  |
|  |  |  |  |  |  |  |  |  |  |  |  |  |  |  |
|  |  | df | SS | *F* | R^2^ | *p* |  |  |  | df | SS | *F* | R^2^ | *p* |
|  | Time | 2 | 3.36 | 27.22 | 0.42 | **0.001** |  |  | Time | 2 | 1.81 | 8.3 | 0.36 | **0.001** |
|  | Temp. | 2 | 1.49 | 12.3 | 0.19 | **0.001** |  |  | Sal. | 1 | 0.59 | 5.4 | 0.12 | **0.001** |
|  | Time*Temp. | 4 | 1.43 | 5.81 | 0.18 | **0.002** |  |  | Time*Sal. | 2 | 0.98 | 4.49 | 0.2 | **0.001** |
|  | Residuals | 27 | 1.66 |  | 0.2 |  |  |  | Residuals | 15 | 1.63 |  | 0.32 |  |
